# Decline of placental malaria in southern Ghana after the implementation of intermittent preventive treatment in pregnancy

**DOI:** 10.1186/1475-2875-6-144

**Published:** 2007-11-08

**Authors:** Lena Hommerich, Christa von Oertzen, George Bedu-Addo, Ville Holmberg, Patrick A Acquah, Teunis A Eggelte, Ulrich Bienzle, Frank P Mockenhaupt

**Affiliations:** 1 Institute of Tropical Medicine and International Health, Charité – University Medicine, Berlin, Germany; 2 Presbyterian Mission Hospital, Agogo, Ghana; 3 Dept. of Medicine, Komfo Anoyke Teaching Hospital, School of Medical Sciences, Kwame Nkrumah University of Science and Technology, Ghana; 4 Department of Bacteriology and Immunology, Haartman Institute, University of Helsinki, Helsinki, Finland; 5 Division of Infectious Diseases, Tropical Medicine and AIDS, Academic Medical Centre, Amsterdam, The Netherlands

## Abstract

**Background:**

Intermittent preventive treatment in pregnancy with sulphadoxine-pyrimethamine (IPTp-SP) has been adopted as policy by many countries in sub-Saharan Africa. However, data on the post-implementation effectiveness of this measure are scarce.

**Methods:**

Clinical and parasitological parameters were assessed among women delivering at a district hospital in rural southern Ghana in the year 2000 when pyrimethamine chemoprophylaxis was recommended (*n *= 839) and in 2006 (*n *= 226), approximately one year after the implementation of IPTp-SP. Examinations were performed in an identical manner in 2000 and 2006 including the detection of placental *Plasmodium falciparum *infection by microscopy, histidine-rich protein 2, and PCR.

**Results:**

In 2006, 77% of the women reported to have taken IPTp-SP at least once (26%, twice; 24%, thrice). In 2006 as compared to 2000, placental *P. falciparum *infection was reduced by 43–57% (*P *< 0.0001) and maternal anaemia by 33% (*P *= 0.0009), and median birth weight was 130 g higher (*P *= 0.02). In 2006, likewise, women who had taken ≥ 1 dose of IPTp-SP revealed less infection and anaemia and their children tended to have higher birth weights as compared to women who had not used IPTp-SP. However, placental *P. falciparum *infection was still observed in 11% (microscopy) to 26% (PCR) of those women who had taken three doses of IPTp-SP.

**Conclusion:**

In southern Ghana, placental malaria and maternal anaemia have declined substantially and birth weight has increased after the implementation of IPTp-SP. Likely, these effects can further be increased by improving IPTp-SP coverage and adherence. However, the remnant prevalence of infection in women having taken three doses of IPTp-SP suggests that additional antimalarial measures are needed to prevent malaria in pregnancy in this region.

## Background

Malaria in pregnancy is a major cause of maternal, foetal and infant morbidity and mortality in sub-Saharan Africa. Although frequently asymptomatic, consequences of *Plasmodium falciparum *infection in pregnancy comprise maternal anaemia, abortion, stillbirth, intrauterine growth retardation, low birth weight (LBW), preterm delivery, and up to 200,000 attributable infant deaths per year [1-5]. Primiparae are particularly vulnerable as immune mechanisms preventing placental sequestration of pregnancy-specific parasite strains are weak or absent [6,7].

Intermittent preventive treatment in pregnancy (IPTp) denotes the administration of a curative dose of an antimalarial, commonly sulphadoxine-pyrimethamine (SP), during routine antenatal care, irrespective of parasitaemia being present or not. Given twice or thrice in the second and third trimester of pregnancy, IPTp-SP has been shown to be safe and to reduce placental malaria, maternal anaemia, and LBW [8-13]. IPTp with ≥2 doses of SP is currently recommended by the World Health Organisation [14], and has become policy in much of sub-Saharan Africa. Nevertheless, coverage with IPTp still needs to be substantially improved [15,16] and the effectiveness of IPTp may be endangered by spreading and intensifying SP resistance [17,18]. Although most clinical trials on IPTp-SP were conducted in East Africa [8-10,19] the strategy was rapidly adopted also in West Africa where its effectiveness is less well documented [20]. Consequently, there is an urgent need to monitor the post-implementation effectiveness of IPTp in this region.

In southern Ghana, malaria in pregnancy and related morbidity are frequent [21-23]. Ghana started the implementation of IPTp with three recommended doses of SP at the end of 2004. In this country, SP achieves cure rates within 28 days of follow-up of 14% and 11% in children and pregnant women with uncomplicated malaria, respectively [24,25]. Here, parasitological and clinical parameters were compared among women delivering at the Presbyterian Mission Hospital in Agogo, southern Ghana, before (2000) and after (2006) the implementation of IPTp. In addition, these parameters were examined with respect to the usage of IPTp among women delivering in 2006 and the number of doses taken.

## Methods

Agogo is a community of some 30,000 inhabitants; subsistence farming, trade and mining are the main income sources in that region, and malaria is hyper- to holoenedemic [26]. The characteristics of 839 women with live singleton delivery recruited in 2000 have been described in detail elsewhere [23]. Between April and June 2006, i.e. during the rainy season, further 226 women with live singleton delivery were recruited. Both study protocols were reviewed and approved by the Ethics Committee, University of Science and Technology, Kumasi, and all women provided informed written consent. Sample collection as well as clinical and parasitological examinations were performed in an identical manner in 2000 and 2006 and have previously been described [23]. In brief, women were clinically examined and socio-demographic data documented. Information on chemoprophylaxis or IPTp-SP was collected from antenatal care cards and verified by interviewing the women. Venous peripheral and placental blood samples were collected into EDTA. Malaria parasites were counted microscopically on Giemsa-stained thick blood films per 500 white blood cells (WBCs) for peripheral samples and per 100 high-power fields (1,000x) for placental samples. In the latter, the presence of leukocyte-associated haemozoin was also recorded. Slides were declared negative after having examined microscopic fields corresponding to 500 WBCs, or 100 high-power fields. Based on placental thick blood film microscopy, the stage of placental infection was categorized [27] as early, only parasites visible; late, both parasites and pigment present; resolved, only pigment visible; and none, neither parasites nor haemozoin present. In placental samples, *P. falciparum *was additionally diagnosed by detection of histidine-rich-protein 2 (HRP2) and nested PCR assays [28]. In 2000, HRP-2 was measured using the ICT Malaria P.f/P.v (Becton Dickinson, Germany) and, in 2006, using Malaria NOW P.f./P.v. (Inverness Medical, Germany). Haemoglobin (Hb) was measured by a HemoCue photometer (Ångelholm, Sweden) and anaemia and severe anaemia defined as Hb < 11 and < 7 g/dL, respectively. Fever was defined as an axillary temperature ≥ 37.5°C and LBW as a birth weight < 2500 g.

For subsequent comparisons, three groups were formed, i) 226 women recruited in 2006, ii) 839 women recruited in 2000, and iii) 197 women who delivered in 2000 during a period exactly matching the recruitment phase in 2006 (29.04.-27.06.). Geometric mean parasite densities (GMPDs) and 95% confidence intervals (95%CIs) were calculated. Continuous variables were compared between groups by the non-parametric Mann-Whitney U and Kruskal-Wallis tests as applicable, and proportions by χ^2 ^test, Fisher's exact test and logistic regression models.

## Results

The baseline characteristics of the three groups of delivering women are shown in Tables 1 and 2. In 2000, virtually all women stated to have used pyrimethamine (25 mg weekly) for chemoprophylaxis during pregnancy. In 2006, 76.5% (173/226) of women reported to have taken IPTp-SP at least once, one women had used chloroquine chemoprophylaxis, and 23.0% (52/226) neither IPTp nor chemoprophylaxis. Eight percent of the women (17/208) in 2006 reported to have slept under a bed net the night before delivery.

**Table 1:** Characteristics of women with live singleton delivery at Agogo Hospital, southern Ghana, in 2000 and 2006

	Year of delivery				
			
Parameter	2000	2000, matching dates subset	2006	2006 *vs*. 2000, *P*	2006 *vs*. 2000 *subset, P*
No.	839	197	226	-	-
Residence in Agogo (%)	48.9	50.3	42.5	0.09	0.13
Age (years; median, range)	25 (15–47)^a^	25 (16–42)^b^	26 (16–42)	0.12	0.31
Proportion without school degree (%)	15.4 (128/829)	13.8 (27/195)	10.4 (23/222)	0.06	0.27
Parity (median, range)	2 (1–11)	2 (1–9)	2 (1–11)	0.14	0.43
Primiparae (%)	36.5 (304/832)	34.2 (66/193)	32.7	0.29	0.75
Proportion caesarean section (%)	22.1	18.3	24.8	0.38	0.11
No. of ANC visits (mean, range)	3.9 (0–12)	3.9 (0–11)	4.5 (0–14)	**0.01**	**0.047**
Proportion with > 3 ANC visits	52.3 (429/821)	48.7 (93/191)	58.7 (131/223)	0.08	**0.04**
Birth weight (g; median, range)	2,950 (1,280–4,500)^c^	2,900 (1,450–4,350)	3,080 (1,600–5,460)^d^	**0.02**	**0.0008**
LBW (%)	16.0 (134/838)	19.3	12.4 (28/225)	0.19	0.05
Haemoglobin (g/dL; median, range)	11.5 (4.6–16.8)	11.7 (4.6–15.0)	12.0 (7.2–15.9)	**< 0.0001**	**0.04**
Maternal anaemia (%)	35.2	33.0	23.5	**0.0009**	**0.03**
Severe anaemia (Hb < 7; %)	1.8	1.5	0	0.05	0.10
Fever (%)	2.8 (23/826)	1.5 (3/196)	6.6	**0.006**	**0.01**

ANC, antenatal care; LBW, low birth weight; ^a^, *n *= 827; ^b^, *n *= 192; ^c^, *n *, = 838; ^d^, *n *= 225

**Table 2:** Parasitological indices of delivering women at Agogo Hospital, southern Ghana, in 2000 and 2006

	Year of delivery				
			
Parameter	2000	2000, matching dates subset	2006	2006 *vs*. 2000, *P*	2006 *vs*. 2000 *subset*, *P*
No.	839	197	226		
Placental infection (%)					
Microscopy	34.9	29.9	15.0	**<0.0001**	**0.0002**
Pigment	30.9	31.0	17.7	**<0.0001**	**0.001**
HRP2	40.8	38.6	22.1	**<0.0001**	**0.0002**
PCR	59.4	51.3	31.9	**<0.0001**	**<0.0001**
Stage of placental infection					
Resolved	17.9 (64/357)	28.1 (23/82)	27.7 (13/47)		
Late	54.6 (195/357)	46.3 (38/82)	57.4 (27/47)		
Early	27.5 (98/357)	25.6 (21/82)	14.9 (7/47)	0.10	0.31
Placental parasite density (GMPD, 95%CI)	0.78 (0.57–1.03)	0.61 (0.32–1.15)	0.31 (0.12–0.80)	0.06	0.23
Peripheral blood film positive (%)	19.0	11.2	14.6	0.13	0.29
Peripheral parasite density (GMPD, 95%CI)	558 (404–772)	506 (224–1142)	891 (366–2172)	0.24	0.39

HRP2, histidine rich protein 2, GMPD, geometric mean parasite density; 95%CI, 95% confidence interval

As compared to 2000, women in 2006 more frequently had attended antenatal care, tended to be older, less likely to originate from Agogo, and more commonly to have a school degree. Maternal anaemia was reduced by 33% in 2006, severe anaemia absent, and median Hb (+0.5 g/dL) and median birth weight (+130 g) were higher. Fever occurred more often in 2006 than in 2000 (Table 1). The prevalence of microscopically confirmed placental parasitaemia was more than halved (reduction by 57%) in 2006 as compared to 2000, and parasite density reduced at boderline statistical significance. Similar reductions in the prevalence of infection were observed considering the results of pigment detection (by 43%), HRP2 tests, and PCR (both by 46%). The stage of placental infection, peripheral blood film positivity and peripheral parasite densisty were not significantly changed, however (Table 2). Comparing these parameters between women delivering in 2006 and women delivering during the matching period in 2000, basically the same results were seen (Tables 1 and 2).

Stratifying by parity, improvements in haematological and parasitological parameters in 2006 as compared to 2000 were found to be more pronounced in multiparae than in primiparae (Table 3). In contrast, there was a considerable reduction in the rate of LBW among primiparae but hardly a change in multiparae. Adjusting the 2000 data set for the months of recruitment in 2006 diminished these parity-dependent differences, particularly with respect to placental infection (Table 3), whereas regard of potential confounders (age, residence, antenatal care, educational status) did not have such an effect.

**Table 3:** Clinical and parasitological indices in delivering women according to parity and year of delivery

Parameter	Primiparae				Multiparae			
		
	2000	2006	aOR (95%CI)	*P*^†^	2000	2006	aOR (95%CI)	*P*^†^
No.	304	74			528	152		
Hb (g/dL; median, range)	11.4 (4.6–16.4)	11.6 (7.7–14.8)	-	0.11	11.6 (4.7–16.8)	12.1* (7.2–15.9)	-	0.08
Maternal anaemia (%)	38.5	35.1	0.74 (0.37–1.46)	0.38	33.5	17.8*	0.55 (0.31–0.96)	**0.04**
Birth weight (g; median, range)	2,785 (1,280–4,000)	2,900* (1,600–4,600)	-	**0.006**	3,020 (1,500–4,500)	3,130 (1,650–5,460)	-	**0.02**
LBW (%)	26.0	16.2	0.41 (0.19–0.93)	**0.03**	10.2 (54/527)	10.6 (16/151)	0.82 (0.39–1.72)	0.60
Placental infection (%)								
Microscopy	46.1	25.7*	0.44 (0.22–0.90)	**0.02**	28.4	9.9*	0.39 (0.20–0.76)	**0.006**
Pigment	42.4	27.0*	0.44 (0.22–0.90)	**0.02**	24.2	13.2*	0.49 (0.26–0.91)	**0.02**
HRP2	50.7	39.2	0.57 (0.29–1.12)	0.10	35.2	13.8*	0.35 (0.19–0.63)	**0.0005**
PCR	65.1	44.6*	0.46 (0.23–0.91)	**0.03**	56.4	25.7*	0.41 (0.25–0.68)	**0.0005**

aOR, odds ratio adjusted for months of recruitment in 2006; 95%CI, 95% confidence interval; *, bivariate comparison 2000 *vs*. 2006; *P *< 0.05 ^†^, *P *value derived from logistic regression models, or from multiple linear regression models (coding: 0, 2000; 1; 2006; and 0, non-matching recruitment period; 1, matching recruitment period)

Next, women delivering in 2000 were compared with the small group of women delivering in 2006 who had not taken IPTp. Placental *P. falciparum *infection was non-significantly reduced by 12% to 17% in 2006 with the exemption of microscopically proven parasitaemia which dropped by 40% (*P *= 0.03; Tables 2 and 4). Birth weight and Hb as well as the proportions of LBW and anaemia were very similar in these two groups (Tables 1 and 4).

**Table 4:** Clinical and parasitological indices in delivering women in 2006 according to intermittent preventive treatment in pregnancy

Parameter	Intermittent preventive treatment in pregnancy				
	
	None^a^	All	One dose	Two doses	Three doses
No.	53	173	60	59	54
Age	24 (17–42)	27 (16–41)*	26 (16–40)	26 (16–40)	28 (16–41)*
Primiparae (%)	41.5	30.1	35.0	27.1	27.8
Proportion with > 3 ANC visits (%)	35.3 (18/51)	65.7* (113/172)	41.7	65.5* (38/58)	92.6*
Birth weight (g; median, range)	2,900 (1,820–4,000)	3,100 (1,600–5,460)	3,025 (2,200–5,460)	3,100 (1,600–4,250)	3,125 (2,030–4,100)
LBW (%)	15.4 (8/52)	11.6	10.0	15.3	9.3
Hb (g/dL; median, range)	11.5 (7.2–14.5)	12.1 (7.7–15.9)*	12.1 (8.4–15.6)*	11.9 (7.7–14.4)	12.4 (9.3–15.9)*
Maternal anaemia (%)	39.6	18.5*	16.7*	27.1	11.1*
Fever (%)	1.9	8.1	13.3*	5.1	5.6
Placental infection (%)					
Microscopy	20.8	13.3	13.3	15.3	11.1
Pigment	26.4	15.0	15.0	15.3	14.8
HRP2	35.8	17.9*	13.3*	22.0	18.5*
PCR	49.1	26.6*	21.7*	32.2	25.9*

^a^, includes one women with previous chloroquine chemoprophylaxis; ^b^, median, range; *, as compared to women without IPTp, *P *< 0.05

Lastly, clincial and parasitological parameters of women delivering in 2006 were analysed according to the previous administration of IPTp (Table 4). All women previously on IPTp had attended antenatal care at least once, but also 80% (41/51) of those who had not taken IPTp (*P *< 0.0001). Overall, 23.5%, 26.5%, 26.1%, and 23.9% of the women had received none, one, two, and three doses of IPTp, respectively. The number of IPTp doses and antenatal care attendance were associated (Table 4). The proportion of women having used IPTp was similar in individuals from Agogo (79.2%) and elsewhere (74.6%; *P *= 0.42) as were the number of doses taken (both groups, median 2, range 1–3; *P *= 0.54). Primiparae (70.3%) and multiparae (79.6%) did not differ significantly in the use of IPTp (*P *= 0.12), or in the number of doses taken (both groups, median 2, range 1–3; *P *= 0.42). The median gestational age (range) at IPTp doses one, two, and three was 24 (16–39), 28 (18–38), and 32 (22–37) weeks, respectively. The gestational week at the first IPTp administration was highest among women who had received one dose only (28.5, 19–39), and lower in women who had taken two (24, 16–32) or three doses (20, 16–24; *P *< 0.0001).

Overall, women who had received IPTp exhibited significantly less placental malaria and maternal anaemia than women who had not used IPTp; also, the former were older and had higher Hb concentrations (Table 4). Parasite densities did not differ between women with and without previous IPTp (data not shown). In women with three preceding doses of IPTp, birth weight and Hb concentrations were highest and anaemia least common. However, there was no clear-cut trend for less morbidity or placental malaria with increasing number of IPTp doses administered (Table 4).

## Discussion

In this observational study, women delivering at a district hospital in rural southern Ghana after the implementation of IPTp-SP had significantly less placental malaria and anaemia and babies of higher birth weight than six years before when pyrimethamine chemoprophylaxis was the common mode of malaria prevention. This is a substantial improvement which, however, cannot exclusively be attributed to the implementation of IPTp since further factors known to be or potentially involved, e.g. antenatal care attendance, residence, and educational status, also changed over time. A further limitation of the present study is that malaria incidence or transmission intensity in Agogo and surroundings could have declined between 2000 and 2006. The implementation of e.g. artemisinin-based combination treatment in Ghana and other interventions might have led to a reduced malaria transmission and burden of disease. However, no dependable data are available to substantiate this. Though, in women in 2006 who did not take IPTp, the prevalence of placental infection (but not of LBW or anaemia) was lower than in 2000. However, this reduction did not have the magnitude seen in the 2006 group overall or among women having used IPTp. Moreover, comparing women delivering in 2006 with and without previous IPTp points into the same direction suggesting that IPTp in fact has markedly reduced the burden of malaria in pregnancy and related consequences. Still, 32% of women delivering after the implementation of IPTp were found to be *P. falciparum *infected, and maternal anaemia and LBW occurred in 24% and 12%, respectively, with higher figures among primiparae. This likely results from both, incomplete IPTp coverage and a remnant burden of disease which cannot further lowered even by complete coverage with this one measure of malaria control.

Data on IPTp-SP in West Africa are rare. In Mali and as compared to chloroquine chemoprophylaxis, 2-dose IPTp-SP significantly reduced the risks of placental parasitaemia, maternal anaemia, and LBW [11]. In a smaller trial from Nigeria, IPTp-SP was superior to pyrimethamine chemoprophyaxis in the prevention of parasitaemia and anaemia which was confirmed and complemented by a beneficial effect on birth weight in a recent observational study [29,30]. In Burkina Faso, the implementation of IPTp-SP in one district reduced placental parasitaemia and LBW, the latter, however, only when three doses had been taken [31]. Lastly, in The Gambia, IPTp-SP benefited multigravidae only when not protected by a bednet [32]. Altogether, these data show that IPTp-SP in West Africa is superior to available chemoprophylactic approaches but the effects achieved vary geographically and with the characteristics of the target population.

In the present study, only a quarter of delivering women had received all three recommended doses of IPTp-SP, and only half had taken two or more doses. Antenatal care attendance and both, use of IPTp-SP and number of doses were associated illustrating that efforts to promote antenatal care will likely be paralleled by increasing IPTp coverage, and, consequently, lead to even more pronounced effects than observed here. At Agogo hospital, the first dose of IPTp-SP was taken at a median gestational age of 24 weeks, i.e. roughly at halftime of pregnancy. While observed treatment during antental care visits is a major asset of IPTp, its comparatively late utilisation during pregnancy in general is a major drawback. Malaria early in pregnancy not only contributes to abortion but also to intrauterine growth retardation and LBW [2]. So far, it is unknown whether the pathophysiological changes and foetal damage induced by infections in early pregnancy are reversible and, thus, the extent of morbidity due to the relatively late initiation of IPTp cannot be estimated. Education and information campaigns flanking IPTp as well as community-based delivery systems may promote early initiation of IPTp and also improve access, attendance and adherence to the programme [33,34]. Further measures of malarial control, however, are needed to cover the vulnerable period of early pregnancy. Insecticide treated nets (ITNs) are a suitable and effective option [35], but the bed net coverage rate (8%) seen in this study is far too low.

Primiparae in 2006 showed an only slightly lower rate of IPTp usage than multiparae arguing for a similar level of information and acceptance among women of different parities. Elsewhere, however, primiparity, young age, and, thereby, a presumed low level of malaria-related knowledge, might be an obstacle in taking up interventions such as IPTp. IPTp should be most effective in primiparae in whom antimalarial immunity is lowest and infection rates are highest [1,4,6,7,23]. In fact, data from East Africa and from multigravidae in The Gambia point into this direction [8,32]. Adjusting for the recruitment period, the reductions in placental malaria observed between 2000 and 2006 were similar in primi- and multiparae but the the reduction in maternal anaemia was significant only in the latter. The proportion of LBW, in contrast, declined significantly only in primiparae. Further adjustment for potential confounders did not fundamentally change these findings. These parity-dependent differences could reflect a differential effect of reducing the prevalence of malaria: in multiparae, increasing Hb concentrations might be the most visible sign of improved malaria prevention whereas the contribution of malaria to LBW is comparatively small [36]. In fact, the rate of LBW among multiparae in this study closely resembles the figure among African Americans [37]. In contrast, in primiparae the proportion of malaria-attributable LBW is larger than in multiparae [36], and IPTp-SP thus can achieve a greater extent of LBW reduction. So far, it is unclear why primiparae did not benefit from IPTp in haematological terms. Beyond a comparatively small sample size, this could stem from the still high prevalence of placental malaria in this group, or alternatively, reflect the importance of other, non-malaria causes of anemia. Such could involve e.g. iron deficiency which has previously been observed in only 5–18% of pregnant women in Agogo [22] and HIV infection, which occurs among 3% of pregnant women in Ghana [38].

No straightforward trend for less malaria, anaemia or LBW with increasing number of IPTp doses was observed in this study. Stratification into relatively small subgroups might be one reason. The sample size also impeded a meaningful analysis of the effects by parity and of the time when the last IPTp dose was given. The latter has been shown to unevenly influence the risk reduction of placental malaria in Kenya [39]. Selection of drug-resistant parasites during the course of IPTp-SP could also be involved but, so far, it is not understood whether and to which extent this occurs. In children, a substantial increase in the proportion of resistant parasites has been observed within weeks after preventive SP treatment [40].

Drug resistance might also be responsible for the prevalence of 26% of placental *P. falciparum *infection observed in women who had taken all three doses of IPTp-SP. Already in 2000, 52% of placental *P. falciparum *isolates from Agogo hospital exhibited the triple *dihydrofolate reductase *mutation (Ile51+Arg59+Asn108) [41] which in Ghanaian children increases the risk of SP treatment failure ten-fold [42]. Preliminary data from a subset of the study participants indicate that this figure has increased to more than 75% in 2006. While these findings support the utilisation of preventive approaches in addition to IPTp-SP they also question the useful lifespan of IPTp-SP in the study area and support the urgent evaluation of alternative drugs.

## Conclusion

In rural southern Ghana, the prevalences of placental malaria and of maternal anaemia have substantially been reduced following the implementation of IPTp-SP, and birth weight has increased. These achievements may be improved by increasing the proportion of pregnant women receiving IPTp-SP *via *enforced antenatal care services and information campaigns. Even then, however, a remnant prevalence of malaria in pregnancy will probably remain necessitating the implementation of further means of preventing malaria in pregnancy. Meanwhile, alternative options to SP in IPTp should urgently be evaluated.

## Authors' contributions

FPM, GBA, TAE and UB designed the study. LH, CvO, VH, PAA, and FPM were responsible for patient recruitment, and clinical and parasitological examinations. LH and FPM did the statistical analyses. LH, TAE and FPM wrote the paper with major contributions of the other authors.
